# Joint symbolic dynamics for the assessment of cardiovascular and cardiorespiratory interactions

**DOI:** 10.1098/rsta.2014.0097

**Published:** 2015-02-13

**Authors:** Mathias Baumert, Michal Javorka, Muammar Kabir

**Affiliations:** 1 School of Electrical and Electronic Engineering, University of Adelaide, Adelaide, SA 5005, Australia; 2 Department of Physiology, Jessenius Faculty of Medicine, Comenius University, Martin, Slovakia; 3 Knight Cardiovascular Institute, Oregon Health and Science University, Portland, OR 97239, USA

**Keywords:** symbolic dynamics, heart rate, blood pressure, respiration, baroreflex, respiratory sinus arrhythmia

## Abstract

Beat-to-beat variations in heart period provide information on cardiovascular control and are closely linked to variations in arterial pressure and respiration. Joint symbolic analysis of heart period, systolic arterial pressure and respiration allows for a simple description of their shared short-term dynamics that are governed by cardiac baroreflex control and cardiorespiratory coupling. In this review, we discuss methodology and research applications. Studies suggest that analysis of joint symbolic dynamics provides a powerful tool for identifying physiological and pathophysiological changes in cardiovascular and cardiorespiratory control.

## 1. Introduction

The cardiovascular and respiratory systems are crucial for maintaining blood perfusion and oxygenation of human body tissues. Their parameters are intricately controlled to correspond to the changes in the metabolic demand. This is achieved by a complex network of receptors, afferent pathways, integration of sensory input and efferent hormonal and neural pathways [1]. Observation of cardiovascular and respiratory variables and their interplay allows inferring information on underlying control mechanisms [2].

A significant amount of research has been dedicated to the processing of cardiovascular and respiratory signals to derive clinically meaningful markers of dysfunction in their control systems. In particular, beat-to-beat time series of heart period (i.e. RR interval in the electrocardiogram; ECG), systolic arterial pressure (SAP) and respiratory rate or phase, respectively, have been studied extensively. In most of those studies, linear shift-invariant systems approaches have been adopted to measure the relationship between signals, and time- and frequency-domain representations were chosen to characterize signals and underlying control systems [3]. While these models have yielded important findings across a large number of studies [4,5], the inherent nonlinearity of biological systems imposes significant limitations and often proved them inadequate for characterizing complex dynamics [6,7]. Consequently, alternative approaches have been proposed that capture nonlinear features [8].

Symbolic dynamics is a powerful nonlinear approach that involves coarse-graining of observed time series into sequences of symbols (‘words’) by partitioning the system's phase space into few symbols and capturing the system's trajectory. Although detailed information is lost in the process, significant patterns emerge that can be used to quantify system dynamics. Symbolic dynamics has been extensively explored to characterize dynamics in RR interval time series in health and disease, and its relevance in the field of heart rate variability (HRV) research has been firmly established [9,10]. In humans, the percentage of specific symbolic patterns follows the gradual sympathetic activation and vagal withdrawal induced by graded head-up tilt test [11] and the circadian rhythm of autonomic function [12]. Different symbolization strategies for HRV analysis have been discussed elsewhere [13].

Extending the framework of univariate symbolic dynamics, analysis of joint symbolic dynamics (JSD) has been proposed for the study of interactions between cardiovascular variables over a decade ago [14]. Based on the original works of symbolic dynamics for HRV analysis, multivariate data are individually coarse-grained into symbol sequences and their joint occurrences are quantified [14–16]. By incorporating *a-priori* knowledge about underlying physiological processes in the coarse-graining procedure, a simple yet effective description of clinically relevant phenomena is achieved.

### (a) Joint dynamics of blood pressure and heart rate

Under normal resting conditions, heart period and SAP time series display closely related dynamics, and their behaviour is jointly studied to infer information on cardiovascular control [17]. RR and SAP influence each other in a closed-loop fashion. Heart rate (HR) drives blood pressure via haemodynamic effects, while SAP provides feedback to HR via the cardiac baroreflex that maintains short-term control of blood pressure by modulating the sinoatrial node activity via the vagal and sympathetic pathways [17]. Drops in SAP are counterbalanced by shortening of RR, while increase in SAP results in a prolongation of RR. The responsiveness of HR to blood pressure changes is measured by various indices of the so-called baroreflex sensitivity (BRS) that relate the magnitude of change in RR to the magnitude of change in SAP. Traditionally, pharmacological intervention has been carried out to provoke cardiac baroreflex responses, while alternative approaches aim to measure baroreflex function based on spontaneous fluctuations in SAP, i.e. ‘spontaneous BRS’. Advantages and disadvantages of both approaches have been discussed elsewhere [18].

Traditional quantification of the SAP–RR relationship is based on cross-power spectral analysis that allows one to estimate gain and phase relationships between SAP and RR. A commonly used method to estimate BRS involves computing the square-root of the power ratio of SAP and RR evaluated in the high- and low-frequency ranges where the squared coherence function is larger than 0.5 (alpha index). In the time domain, short sequences of decreasing SAP paralleled by decreasing RR and vice versa are used to estimate the slope as a measure of BRS (‘sequence method’) [19]. More recent approaches for baroreflex assessment are based on Granger causality and allow disentangling feedforward from feedback directions, using multivariate autoregressive models [3] or conditional entropy-based estimation of information transfer between RR and SAP time series [20]. Additionally, these methods are able to measure a degree of signals' synchronization in both directions separately.

From a clinical point of view, BRS assessment is of importance, since reduced BRS has been associated with reduced cardiovascular fitness and increased cardiac morbidity and mortality [21]. In trials involving patients after acute myocardial infarction, reduced BRS was shown to be predictive of sudden cardiac death [22].

### (b) Joint dynamics of respiration and heart rate

The association between cardiac and respiratory rhythms has long been recognized [23–25] and comprises three different phenomena. Firstly, respiratory sinus arrhythmia (RSA) [26] is characterized by cyclic oscillation of HR, with acceleration during the inspiratory phase and deceleration during expiration [27]. RSA is vagally mediated, with inputs to cardiac vagal neurons both from the central pattern generator [28] and from peripheral receptors [29]. Secondly, cardiorespiratory phase coordination or synchronization, respectively, is another phenomenon that was initially described as short intermittent periods [23,30,31] during which the phases of cardiac and respiratory cycle coincide with different integer ratios known as phase locking ratios [24,25,32]. Rodent studies suggest that phase synchronization/coordination is mediated by excitatory effects from arterial baroreceptors to the central respiratory pattern generator [33]. Finally, cardioventilatory coupling (CVC) is another phenomenon that specifically refers to the influence of timing of breathing on cardiac activity [34–36]. CVC is illustrated by temporary alignment between the R waves of the ECG and inspiratory onsets, using the R-peak to inspiratory onset interval plot. During anaesthesia, heart beats and inspiratory onsets are aligned such that they are maximally affected by vagal modulation of RSA, implying common physiological roles and a significant relationship between CVC and RSA [37]. The physiological significance of various aspects of cardiorespiratory interaction is yet to be elucidated. From a clinical point of view, the quantification of cardiorespiratory interaction seems to have merit for risk stratification of cardiac mortality [38–41], and diagnosing the obstructive sleep apnoea syndrome [42–44].

## 2. Methods

Conceptually, symbolic dynamics refers to partitioning a system's phase space into a discrete set of symbols. The trajectory of a system is consequently expressed as a sequence of symbols (‘words’), where the length of the sequence is directly related to the dimension of the phase space. Using Taken's embedding theorem [45], the phase space of a system can be reconstructed by delayed embedding of an observed variable of the system. Delayed phase-space embedding requires knowledge of the system's dimensionality as well as correlation within the time series and, unless *a-priori* knowledge about the system exists, estimating these properties from observed data. Although tools for estimating dimensionality and correlation exist, phase-space reconstruction using biomedical signals has proved challenging [46]. Symbolic analysis of cardiovascular and cardiorespiratory dynamics therefore usually adopts a rather pragmatic approach, where symbolization and dimension (i.e. word length) are defined based on *a-priori* knowledge about the system, features of interest and the amount of available data. Joint analysis of symbolic dynamics requires additional consideration of potential phase lag between time series.

### (a) Symbol transformation

Owing to the limited amount of beat-to-beat cardiovascular and cardiorespiratory data that can be recorded for most experimental conditions, symbolization of time series has been restricted to binary and tertiary schemes.

#### (i) Binary symbolization

Binary symbolization has been adopted to encode beat-to-beat changes in bivariate time series *Z*={[*x*_*n*_,*y*_*n*_]^T^}_*n*=0,1,…_,*x*,*y*∈*R*, into a bivariate symbol sequence *S*={[*a*_*n*_,*b*_*n*_]^T^}_*n*=0,1,…_,*a*,*b*∈0,1, using the transformation rules [14]:
2.1
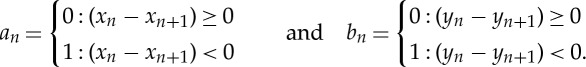

Thus, the embedding delay is one. Binary symbolization provides a very compact representation of variability in the time series that captures ordering of beat-to-beat increases and decreases, respectively, but is sensitive to noise and biased towards symbol ‘0’, which includes zero beat-to-beat changes. Assuming that data have been recorded with high amplitude resolution, the bias might be considered negligible. Alternatively, a non-zero threshold may be included in the symbol transformation, but this effectively excludes some data from the symbolization into *S*.

#### (ii) Tertiary symbolization

Tertiary symbolization is given by the following rules:
2.2
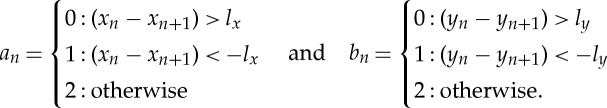

These add an additional element to the set of *S*. This allows distinguishing high beat-to-beat variability beyond defined threshold values *l*_*x*_ and *l*_*y*_ from small changes that might be within the noise floor.

Tertiary symbolization increases the resolution of the symbolic representation of dynamics, but this comes at the expense of word length or constraints with respect to word types if significantly more data are not available.

### (b) Word formation

From the bivariate symbol sequence *S*, short sequences *A* and *B* of length *k* ‘words’ are formed by using a sliding window approach, where *A*=[*a*_*n*_,*a*_*n*+1_,…,*a*_*n*+*k*−1_] and *B*=[*b*_*n*_,*b*_*n*+1_,…,*b*_*n*+*k*−1_]. Leaving aside theoretical concerns regarding the dimension of the system/phase space, the length of words is based purely on statistical considerations, i.e. the amount of available data. Word lengths *k*=3 have been predominantly used in the literature [14].

Considering the binary symbolization scheme, words of length three result in 64 different word types (2^3^⋅2^3^=64), which provide a statistically sufficient representation of dynamics within 30 min recordings of beat-to-beat data on cardiovascular or cardiorespiratory variables. Thus, this approach is able to map the dynamics within four consecutive heartbeats (i.e. three RR intervals). From the perspective of phase-space reconstruction, this corresponds to a three-dimensional embedding, which should be considered a pragmatic solution rather than a true reconstruction of the system's trajectory, but covers the physiologically important time scale of respiratory dynamics. In resting conditions, one respiratory cycle roughly covers four cardiac cycles. The relative frequency of each of the 8×8 combinations of binary symbolic sequences *A* and *B* obtained from the bivariate time series *Z* can be written as a word distribution matrix *W* [14]:
2.3
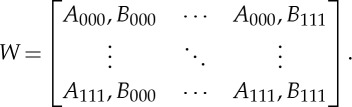

Considering the tertiary symbolization scheme and *k*=3, 3^3^×3^3^=729 combinations of sequences results in a 27×27 word distribution matrix *W* [47]:
2.4
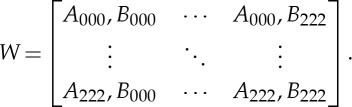

Since 30 min recordings do not yield a statistically robust representation of word types, constraints need to be made as to how *W* is assessed.

#### (i) Accounting for phase shifts between bivariate processes

Although any phase shift between bivariate processes would naturally be reflected in the relative frequency of word types, including *a-priori* knowledge into the temporal alignment between the two processes may yield more meaningful results, in particular because the length of words is rather limited and no true phase-space embedding is realized. An approach for the explicit analysis of time delays between processes in JSD is outlined in §2*e*.

### (c) Joint symbolic analysis for quantifying heart rate and systolic blood pressure dynamics

**Figure 1.**
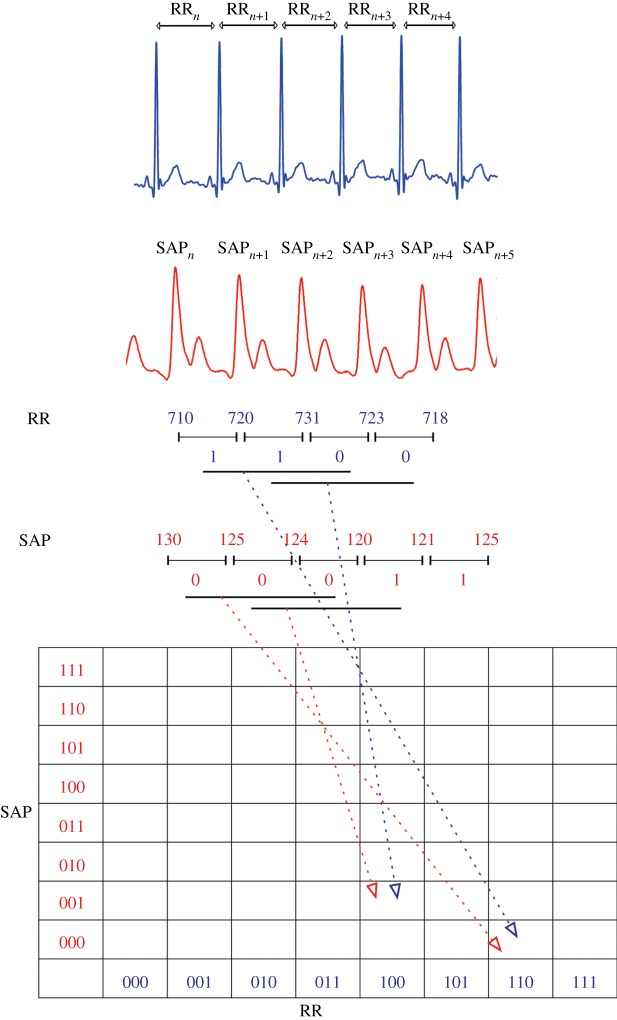
Schematic representation of the joint symbolic analysis of RR interval (RR) and systolic arterial pressure (SAP) dynamics, using a binary symbolization procedure. Beat-to-beat changes in RR and SAP obtained from ECG and continuous blood pressure recordings are transformed to sequences of ‘1’ and ‘0’, encoding increases and decreases (or no change), respectively. Words comprising three symbols are subsequently formed and their relative frequency assessed. (Online version in colour.)

Most studies have employed binary symbolization for the joint analysis of RR and SAP dynamics. Symbolic representations of beat-to-beat changes in RR interval and SAP are aligned such that a change in SAP affects the subsequent RR interval (figure 1). This provides an effective embedding of baroreflex-related RR dynamics.

In addition to assessing the relative frequency of all 64 word types contained in *W*, three indices may be obtained to characterize JSD:

— 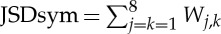, representing symmetric word types,
— 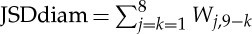, representing diametric word types and
— 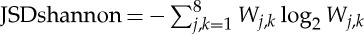, the Shannon entropy of *W*.

JSDsym represents the relative frequency of baroreflex-like word types, while JSDdiam represents the relative frequency of patterns that are opposed to baroreflex behaviour. JSDshannon quantifies the overall distribution of word occurrences and therefore provides a general index of complexity of joint RR and SAP dynamics.

Using a tertiary symbolization approach, a set of indices has been proposed to assess JSD of HR and blood pressure that include zero-variation-in-symbol word types, one-variation-in-symbol word types and alternating symbol sequences [48]. Systematic investigations of appropriate thresholds for tertiary symbolization of RR and SAP dynamics are still lacking. Until then, standard thresholds used for the sequence method of BRS analysis might be applied, where RR changes greater than 5 ms and SAP changes greater than 1 mmHg are considered [19].

### (d) Joint symbolic analysis for quantifying heart rate and respiration dynamics

Initial studies of respiratory and RR dynamics using joint symbolic analysis adopted binary symbol encoding [49]. Since cardiac cycle and respiratory cycle operate at different frequencies, RR and respiratory interval time series were interpolated at 1, 2 or 4 Hz to obtain equidistant time series. Relative frequencies of word types were used to characterize JSD.

Kabir *et al*. proposed a tertiary symbolization scheme and quantified the relative frequency of word types, capturing RSA patterns and thereby adding physiological *a-priori* knowledge to the analysis [47]. To address the issue of different frequencies between cardiac and respiratory oscillators, Hilbert transformation was introduced to obtain the instantaneous respiratory phase (RP) sampled at the R peak in ECG, yielding beat-to-beat symbol sequences of changes in RR interval and respiratory phase. For a discrete signal *x*_*n*_ with *N* samples, the Hilbert transform is defined as
2.5
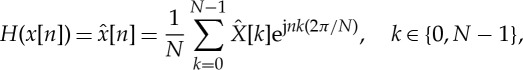

where for even *N*,

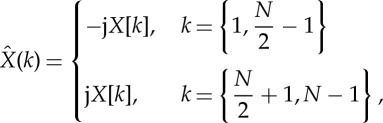

where 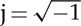 and 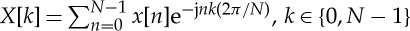, is the discrete Fourier transform. Here, the DC and Nyquist components are excluded (for *k*=0 and *k*=*N*/2). If *N* is odd,

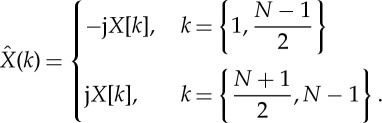

Here, the constant component is excluded.

To account for a potential delay between respiratory phase (*ϕ*_*r*_(*m*); *m*=1,2,3,…,*M*, where *M* is the number of samples) and change in RR interval (*rr*(*i*); *i*=1,2,3,…,*I*, where *I* is the number of RR intervals), the RR time series is shifted with respect to RP in the positive and negative directions such that the correlation between the two sequences is maximized, as determined by the angular–linear correlation coefficient, rRP [50]:
2.6
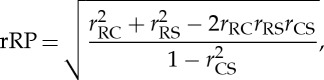

which quantifies association between a linear and an angular variable, where *r*_RC_=*c*(*rr*(*i*+*τ*), cos *ϕ*_*r*_(*m*_*i*_)), *r*_RS_=*c*(*rr*(*i*+*τ*), sin *ϕ*_*r*_(*m*_*i*_)), 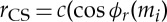, sin *ϕ*_*r*_(*m*_*i*_)) and *τ* represents the RR intervals' delay in beats, *ϕ*_*r*_(*m*_*i*_) represents the respiratory phases at *i*th R peaks and *c*(*u*,*v*) the Pearson correlation coefficient between two variables *u* and *v*. The delays are limited to *τ*=−6,−5,…,0,…,+5,+6 beats, where the negative delays refer to shifting the RR time series by negative *τ* with respect to the respiratory phase.

Symbolization of *rr* and *rp* is carried out using the following rules:
2.7
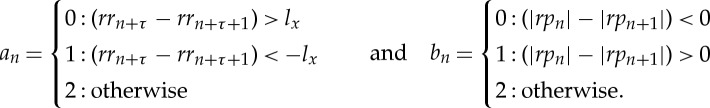

A suitable value threshold value *l*_*x*_ for RR symbolization is 6 ms [47].

### (e) Joint symbolic dynamics for assessing delay and directionality in interaction between time series

The assessment of JSDsym and JSDdiam values as a function of time delay *τ* between time series *x* and *y* has been proposed to assess directionality and delay in interaction between processes [51]. Using the binary symbolization scheme (equation (2.1)) and allowing for variable delay *τ* for the calculation of *b*_*n*_, the transformation rules become:
2.8
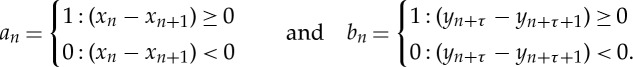

The embedding delay is one and the word distribution matrix *W* is computed for *k*=3 as defined above (equation (2.3)). Subsequently, the proportion of words in diagonals of *W*, i.e. JSDsym and JSDdiam, are computed as a function of *τ*. The difference Δ*T* in the relative frequency of JSDsym and JSDdiam is used to identify directionality and delay in coupling between *x* and *y*:
2.9
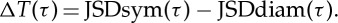

These so-called symbolic coupling traces were shown to effectively separate feedback and feed-forward coupling between RR intervals and SAP [51].

## 3. Joint symbolic analysis of heart rate, blood pressure and respiration in physiology and pathophysiology

### (a) Autonomic function testing

**Figure 2.**
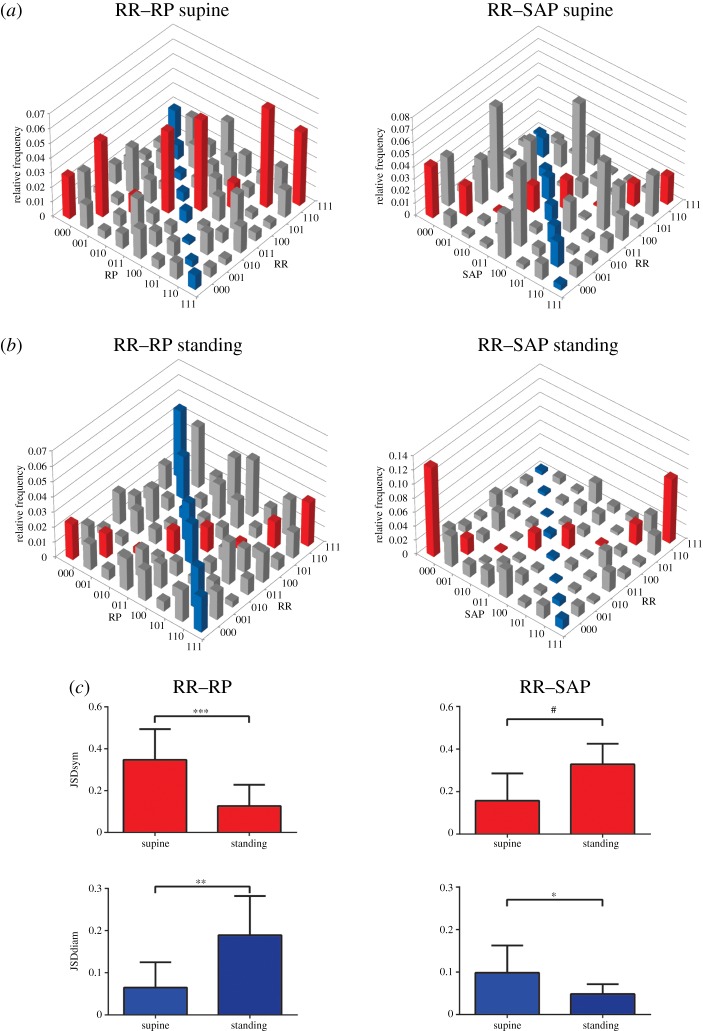
Word distribution matrices of cardiorespiratory (left column) and cardiovascular (right column) symbolic dynamics during rest in the supine position (*a*) and during standing (*b*) as well as relative frequency of symmetric and diametric word types across both conditions (*c*). Data were obtained from 10 healthy athletes (five males/five females). Details have been published elsewhere [55]. It is observed that cardiorespiratory interaction, as quantified by the relative frequency of symmetric word types (JSDsym), decreases upon standing, while the frequency of diametric word types (JSDdiam) increases. By contrast, the relative frequency of JSDsym in joint analysis of HR and SAP increases, while the frequency of JSDdiam word types decreases, which indicates increased baroreflex activity upon standing. Student's *t*-test results are indicated as follows: *p<0.05, ^**^p<0.01, ^***^p<0.001, ^#^*p*<0.0001. (Online version in colour.)

High sensitivity of JSD approaches for quantifying cardiovascular and cardiorespiratory responsiveness to different stress paradigms has been established [52–54]. JSD of RR and SAP show an increase in symmetric baroreflex-like patterns in healthy athletes after postural change from the supine position to active standing (figure 2). Analysis of supine resting RR and SAP dynamics in normal subjects with the symbolic coupling traces method has demonstrated bidirectional coupling with mechanical feedforward direction from RR to SAP at zero lag and baroreflex-related feedback with a delay [56].

Symbolic analysis of cardiorespiratory interaction has helped to document decoupling of RR dynamics from the respiratory phase during orthostatic provocation through active standing as well as during 60^°^ head-up tilt test [47]. Figure 2 shows a decrease in symmetric word types during active standing in healthy athletes, indicative of reduced cardiorespiratory interaction during standing. Joint symbolic analysis of RR, SAP with respect to the respiratory phase showed baroreflex-type RR and SAP dynamics alignment with the respiratory phase during rest in the supine position, but dissociation from respiratory phase during active standing [52,53].

During mental and emotional stress, elicited using the Mannheim multicomponent stress test, an increase in the Shannon entropy of JSD of respiration and RR interval was reported [54]. In rats, mild emotional stress induced by air-jet stress led to a reduction in JSDsym of HR and SAP [57]. Symbolic indices of joint RR and SAP dynamics were shown to correlate with pupillary light reflex [58].

In conscious rats, *α*_1_-adrenergic receptor blockade, β-adrenergic receptor blockade and muscarinic receptor blockade all reduced JSDsym, whereas β-adrenergic receptor blockade increased JSDdiam and muscarinic blockade reduced JSDshannon, demonstrating that joint symbolic analysis of RR and SAP dynamics is sensitive to changes in vagal and sympathetic cardiac activity and sympathetically driven vascular changes [59].

Joint symbolic analysis of RR and SAP during intermittent hypoxia showed reduced interaction in a piglet model of neonatal normocapnic hypoxia while symbolic analysis of RR and respiratory interval time series displayed reduced coupling during reoxygenation, suggesting differentiated responsiveness of cardiorespiratory and cardiovascular control mechanisms to hypoxia [60].

### (b) Ageing

Joint symbolic analysis of HR and SAP dynamics in adults has shown an age-related decoupling of HR from blood pressure as indicated by a reduction in JSDsym that is paralleled by a tendency of JSDdiam to increase [61]. Cardiorespiratory interaction analysis, using JSD, further demonstrated decoupling of HR dynamics from the respiratory phase in elderly subjects [62]. Together, these findings suggest less effective and disconnected cardiovascular control in the elderly.

### (c) Normal sleep and pathology

Several studies have highlighted the effectiveness of JSD approaches to detect changes in autonomic activity during sleep and disturbances caused by sleep disordered breathing. Joint symbolic analysis of RR interval and respiratory interval showed differences in cardiorespiratory dynamics in preterm infants during active versus quiet sleep [63]. In healthy children, symbolic analysis of RR interval and respiratory phase has shown sleep stage-specific dynamics with increased cardiorespiratory interaction during slow wave sleep [64]. Cardiorespiratory interaction in these children was shown to increase acutely after spontaneous arousal from stage 2 sleep [65]. Symbolic analysis of RR and SAP dynamics suggest sleep stage-specific blood pressure control in normal adult subjects and significant changes in patients with obstructive sleep apnoea syndrome [66]. In adult patients with moderate to severe obstructive sleep apnoea syndrome, coupling between RR and respiratory phase was reduced [67] and was highest during slow wave sleep [67,68]. Continuous positive airway pressure therapy resulted in normalization of joint symbolic RR and SAP dynamics during daytime in patients with obstructive sleep apnoea syndrome [69].

### (d) Pregnancy

Pregnancy is associated with substantial changes to the cardiovascular system, which can be quantified using JSD techniques. During normal pregnancy, HR and SAP word distribution was more complex than in non-pregnant women, suggesting reduced baroreflex activity [14]. In pregnancy-induced hypertension, baroreflex activity appears to be reduced even further as indicated by reduced JSDsym and increased JSDdiam word frequencies and JSDshannon [70]. The JSD word distribution matrix also demonstrated differences in pregnancies with chronic hypertension compared with normal pregnancies [71]. Joint symbolic analysis of RR and SAP may indeed be useful for distinguishing preeclampsia from other hypertensive conditions, i.e. pregnancy-induced hypertension and pregnancy with chronic hypertension [72].

### (e) Mental/neurological disorders

Mental and neurological disorders may adversely affect cardiovascular and cardiorespiratory control. Symbolic analysis of RR interval and respiratory phase identified a loss of cardiorespiratory coupling in patients with Parkinson's disease [73]. Joint symbolic analysis of RR and SAP was able to identify impaired cardiovascular control in patients with sensorineural hearing loss [74].

In unmedicated patients with major depressive disorder, JSDsym of RR and SAP dynamics was reduced while JSDdiam and JSDshannon were increased, suggestive of impaired short-term blood pressure control. [75]. Similarly, JSDsym was reduced in medicated patients with major depressive disorder, where patients receiving treatment with serotonin and noradrenaline reuptake inhibitors had lower JSDsym values than patients who were on selective serotonin reuptake inhibitors [76]. This suggests that JSD approaches are able to differentiate effects of antidepressive medication on cardiovascular control.

HR and SAP time series of patients with acute schizophrenia showed reduced JSDsym, increased JSDdiam and JSDshannon, indicative of impaired baroreflex control [77,78]. Differences in the RR and SAP word distribution matrix were also observed between schizophrenic patients, their first degree relatives and normal subjects [79]. Similarly, joint symbolic analysis of respiratory interval and RR interval time series showed increased complexity in patients with schizophrenia [77] and first degree relatives [54] compared with normal subjects. Joint symbolic analysis of RR and SAP based on tertiary transformation suggests impaired blood pressure control of schizophrenic patients compared with normal subjects, and revealed differences between treated and unmedicated schizophrenic patients, pointing towards medication effects on blood pressure control [48]. JSDdiam was increased in alcoholics after acute alcohol withdrawal and captures the impact of alcohol withdrawal on autonomic nervous system activity [80].

### (f) Cardiovascular diseases

In patients with idiopathic dilated cardiomyopathy, word type distribution of HR and SAP dynamics changed from a predominance of baroreflex patterns towards patterns of alternating RR and SAP (i.e. increase–decrease–increase patterns or decrease–increase–decrease patterns) compared to normal subjects, demonstrating impairment in haemodynamic cardiac function [81]. Short-term detrended fluctuation analysis of joint symbolic time series, which provides a measure of long-range correlations, showed a reduction in correlation RR and SAP dynamics in patients with dilated cardiomyopathy compared with normal subjects and indicates a loss of blood pressure control [15]. Changes in the word distribution matrix of RR and SAP dynamics were also observed in patients with persistent atrial fibrillation, who underwent cardioversion to establish sinus rhythm [82]. Here, JSD was predictive of atrial fibrillation recurrence.

### (g) Other conditions

In young patients with type 1 diabetes mellitus, joint symbolic analysis of RR and SAP dynamics showed preserved baroreflex function [83]. Joint symbolic analysis of RR and respiratory rate time series has been employed to predict outcomes in ventilated patients that underwent weaning trials [49,84–86].

### (h) Relationship between joint symbolic dynamics and other indices

Relatively few investigations have been carried out relating indices derived from symbolic dynamics to more established measures of cardiovascular and cardiorespiratory variability. The index JSDsym was shown to be moderately correlated with BRS, as estimated with the sequence method in normal subjects and patients with dilated cardiomyopathy, while JSDdiam showed inverse correlations [15]. In pregnant women and women with pregnancy-induced hypertension, JSDsym was correlated with sequence method based baroreflex indices and JSDshannon displayed inverse correlations [70]. Cardiorespiratory interaction in normal subjects assessed by joint symbolic RR and respiratory phase dynamics was more sensitive to postural change than phase averaged RSA [47].

## 4. Summary

Joint symbolic analysis of cardiovascular and cardiorespiratory dynamics provides a simple yet effective approach to characterize the interaction and control of HR, blood pressure and respiration. It can be considered a complementary method to the mathematical tools derived from the theory of linear shift-invariant systems, which are based on the second-order statistics (e.g. baroreflex gain and delay). Joint symbolic analysis is not restricted to linear relationships. High flexibility in transformation rules and word formation allows encoding and quantifying distinct, physiologically relevant patterns of dynamics that may not be discernible with conventional time and frequency analyses. Although some studies show additive value of symbolic analyses in combination with standard techniques of HRV, BRS and cardiorespiratory coupling analysis, a systematic comparison for establishing clinical research value has not yet been conducted. Suitable threshold values for tertiary symbolization require further investigation.

Although JSD has been primarily used for cardiovascular and cardiorespiratory investigations the methodology is likely to be effective to characterize joint behaviour of other biomedical [87] and non-biological data.
